# Methanolic Extract of *Ficus carica* Linn. Leaves Exerts Antiangiogenesis Effects Based on the Rat Air Pouch Model of Inflammation

**DOI:** 10.1155/2015/760405

**Published:** 2015-04-21

**Authors:** Tahereh Eteraf-Oskouei, Saeideh Allahyari, Arezu Akbarzadeh-Atashkhosrow, Abbas Delazar, Mahdiyeh Pashaii, Siew Hua Gan, Moslem Najafi

**Affiliations:** ^1^Biotechnology Research Center, Tabriz University of Medical Sciences, Tabriz 5166414766, Iran; ^2^Department of Pharmacology and Toxicology, Faculty of Pharmacy, Tabriz University of Medical Sciences, Tabriz 5166414766, Iran; ^3^Student Research Committee, Faculty of Pharmacy, Tabriz University of Medical Sciences, Tabriz 5166414766, Iran; ^4^Department of Pharmacognosy, Faculty of Pharmacy, Tabriz University of Medical Sciences, Tabriz 5166414766, Iran; ^5^Human Genome Centre, School of Medical Sciences, Universiti Sains Malaysia, 16150 Kubang Kerian, Kelantan, Malaysia

## Abstract

The antiangiogenesis effect of* Ficus carica* leaves extract in an air pouch model of inflammation was investigated in rat. Inflammation was induced by injection of carrageenan into pouches. After antioxidant capacity and total phenolic content (TPC) investigations, the extract was administered at 5, 25, and 50 mg/pouch, and then the volume of exudates, the cell number, TNF*α*, PGE_2_, and VEGF levels were measured. Angiogenesis of granulation tissues was determined by measuring hemoglobin content. Based on the DPPH assay, the extract had significant antioxidant activity with TPC of 11.70 mg GAE/100 g dry sample. In addition, leukocyte accumulation and volume of exudate were significantly inhibited by the extract. Moreover, it significantly decreased the production of TNF*α*, PGE_2_, and VEGF, while angiogenesis was significantly inhibited by all administered doses. Interestingly, attenuation of angiogenesis and inflammatory parameters (except leukocyte accumulation) by the extract was similar to that shown by diclofenac. The extract has anti-inflammatory effects and ameliorated cell influx and exudation to the site of the inflammatory response which may be related to the local inhibition of TNF*α*, PGE_2_, and VEGF levels as similarly shown by diclofenac. The antiangiogenesis and anti-VEGF effects of* Ficus carica* may be correlated with its significant antioxidant potentials.

## 1. Introduction

*Ficus carica *Linn. (syn:* Ficus sycomorus*; family: Moraceae) is commonly referred to as “fig.” “Fig” is one of the only five plants mentioned in the holy Quran along with the olives, grapes, pomegranate, and dates. Its fruit, root, and leaves are used in the native system of medicine in different diseases [1]. The tree is one of the oldest plants cultivated in the Mediterranean [2] and has been traditionally used for metabolic, cardiovascular, respiratory, antispasmodic, and anti-inflammatory disorders [1]. It has high minerals, vitamins, dietary fibers, and phenolic contents which play an important role in its antioxidant capacity [3]. Phenolic compounds are common secondary plant metabolites which not only provide important physiological functions in plants but also exert positive effects to the human health [4].

Rheumatoid arthritis (RA) is a chronic and major autoimmune disease that affects many people worldwide [5]. It is an inflammatory condition which usually involves multiple joints and causes functional disability with serious pain [6]. This disorder is also more prevalent among women in developed countries [6, 7]. Synthetic anti-inflammatory agents available today have numerous side effects [8] which give rise to research involved in identification of cheaper and safer alternatives from the natural resources.

A rat model for RA, namely, the air pouch model of inflammation, represents many aspects of joint inflammation. It is a newly formed, bursa-like cavity that grows around subcutaneously injected air and resembles the human synovium. The air pouch has also been used to investigate the effects of acute and granulomatous inflammation on the degradation of juxtaposed cartilage and of antirheumatic and other drugs on this process [9]. It is a readily harvested inflammatory exudate and does not involve internal organs which can be damaged or perforated during sampling [10] and can lead to inaccurate interpretation of findings. Therefore, it has been reported that carrageenan air pouch forms a suitable* in vivo* model to represent the anti-inflammatory activity of a therapeutic agent since it can induce inflammation [11].

The formation of new blood vessels (angiogenesis) is an integral part of the body's physiology [9, 12]. Besides being important in normal bodily function including the period during embryogenesis and menstrual cycle, it also contributes to the pathogenesis of some conditions such as tumor growth, neovascular glaucoma, and RA [9, 13]. Angiogenesis is involved in the early step in RA pathogenesis and has been suggested to be of importance since it leads to leukocyte recruitment and inflammation in the synovium. Furthermore, synovial inflammation itself further potentiates endothelial proliferation in which the expansion of synovial tissue requires a compensatory increase in the number and density of synovial blood vessels. In fact, the arthritic synovium is a very hypoxic environment which is a potent signal for the generation of new blood vessels [13–15]. Even though angiogenesis is a sustaining element for cancers and inflammatory diseases, knowledge on antiangiogenic factors may provide evidences for controlling angiogenesis-dependent diseases including RA [16, 17].

**Figure 1:**
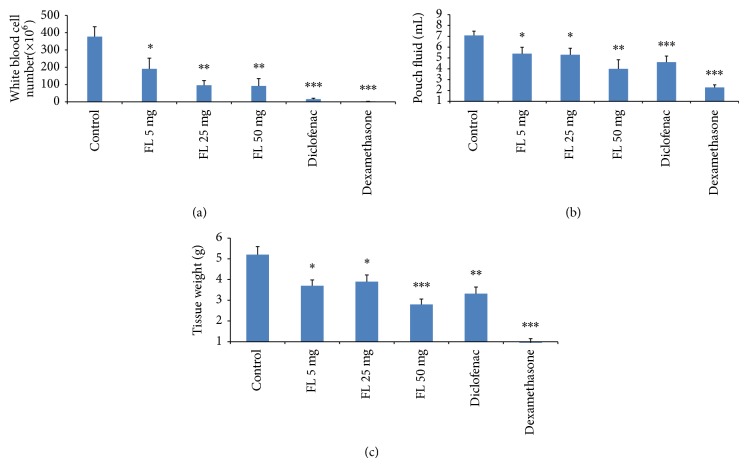
Investigation on the effects of* Ficus carica* extract on the total number of leukocytes (a) in the pouch fluid and (b) on pouch fluid volume and (c) granulation tissue weight, 72 h after carrageenan injection for the different groups. Values are mean with SEM shown by the vertical bars. Asterisks indicate significant difference when compared to the control; ^∗^ *P* < 0.050, ^∗∗^ *P* < 0.010, and ^∗∗∗^ *P* < 0.001 based on ANOVA; FL:* Ficus carica.*

Despite the availability of several reports indicating beneficial effects of fig [1], the antiangiogenic effect of methanolic extract of* Ficus carica* leaves and its impact on the level of inflammatory mediators in air pouch model of inflammation have not yet been investigated. Therefore, the current study was aimed to evaluate anti-inflammatory and antiangiogenic effect of its methanolic extract in this model in rats. We investigated the production of tumor necrosis factor alpha (TNF*α*), as the main regulator of other inflammatory cytokines in the synovial membrane, inhibition of which can pose as a powerful treatment for rheumatoid arthritis [18]. Since proinflammatory prostaglandin E_2_ (PGE_2_) (1) is secreted by many cell types, (2) is the major prostanoid produced by the arthritic synovium, (3) plays important roles in the process of angiogenesis [19–22], and (4) dose-dependently regulates TNF*α* production by macrophages [23], we also determined its levels in this study. In addition, due to the importance of vascular endothelial growth factor (VEGF) as an important regulator of angiogenesis in inflammation [24, 25] and also for validating angiogenesis results, concentrations of VEGF in inflammatory exudates were also determined to further strengthen the findings.

## 2. Materials and Methods

### 2.1. Plant Material and Leaves Extraction

Leaves of* Ficus carica* (100 g) were collected from Azarshahr (East Azerbaijan, Iran). The samples were authenticated by Dr. Delazar from the Pharmacognosy Department, Tabriz University of Medical Sciences, Iran. The leaves were washed, were dried, and were coarsely powdered before storage in a closed metal container. The sample was then extracted by using 100% methanol by means of a soxhlet apparatus followed by drying using an evaporator (Laborota 4002-digital, Germany). It was then stored for a maximum of three months before further analysis.

### 2.2. DPPH Radical Scavenging Assay

A 2,2-diphenyl-1-picrylhydrazyl (DPPH) assay most commonly involves a hydrogen atom transfer. In this experiment, a solution of DPPH (0.08 mg/mL) in methanol was used. The methanolic extract of* Ficus carica* leaves was dissolved in methanol to obtain a concentration of 1 mg/mL. Dilutions were made to yield five different concentrations (1.56 × 10^−2^, 3.13 × 10^−2^, 6.25 × 10^−2^, 12.50 × 10^−2^, and 25.00 × 10^−2 ^mg/mL). Diluted solutions (5 mL each) were mixed with DPPH (5 mL) and were allowed to stand for half an hour at room temperature. The UV absorbance was recorded at 517 nm. The experiment was performed in duplicate and the mean absorption was calculated for each concentration. The concentration that leads to a 50% reduction in absorbance (RC_50_) was calculated [26]. Data were processed using Microsoft Excel (2007).

### 2.3. Total Phenolic Content

Determination of total phenolic content was conducted by Folin-Ciocalteu test according to Heshmati-Afshar et al. (2012) with some slight modifications. Briefly, 1 mL of the extract [5 mg in acetone : water (60 : 40) v/v] was added to 0.2 mL Folin-Ciocalteu's reagent (1 : 2 diluted water) and a 2% sodium carbonate mixture (1 mL). For control, reagent solution alone was used. After 30 min incubation at room temperature, the absorbance was measured at 750 nm. For the calibration curve, 10 mg of gallic acid was dissolved in 10 mL of acetone : water (60 : 40) v/v as a stock solution. The experiments were conducted in duplicate for every dilution to generate the calibration curve [26].

### 2.4. Animals

Male Wistar rats (200–250 g, Pasture Institute, Iran) were housed in standard polypropylene cages, six per cage, under a 12 light: 12 dark schedule. They were provided with suitable environment and nutritional conditions with food and water* ad libitum*. They were acclimatized to the laboratory environment for at least 1 h before investigation and were used only once throughout the study. The animal care and handling procedures were conducted in accordance with the Animal Ethics Guidelines at the Tabriz University of Medical Sciences and “Principles of laboratory animal care” (NIH publication number 86-23, revised 1985).

Two independent sets of experiments were conducted. In the first set, the rats were divided into seven groups of six animals each including a negative control (saline), two positive controls (diclofenac sodium 1.0 mg/pouch and dexamethasone 0.4 mg/pouch, resp.), vehicle-treated group, and* Ficus carica* extract-treated groups at 5, 25, and 50 mg/pouch via intrapouch administration. In the second set of experiments, the rats were divided into 3 groups including control (vehicle-treated) and* Ficus carica* extract-treated (20 and 200 mg/pouch) groups administered with intraperitoneal injections. All animals received intrapouch administration of 1 mL of carrageenan 1% as a phlogistic agent. Stock solutions were diluted with saline and then 1 mL of the diluted solution containing the indicated amount of drugs was injected into the pouch or peritoneum of each rat under light isoflurane anesthesia just before carrageenan injection and for two other consecutive days.

### 2.5. Creation of Air Pouch Type Inflammation by Carrageenan in Rats

Six days prior to induction of inflammation, the rats were anesthetized with low dose isoflurane. The area around the dorsal cervical thoracic region of the animal was shaved and the entire region was swabbed with 70% ethanol followed by subcutaneous injection of 20 mL of sterile air. Three days later, the rats were reanaesthetized and the back of the animals was swabbed with 70% ethanol followed by injection of 10 mL sterile air into the pouch. The second injection of half of the air volume maintained the integrity of the air pouch without causing further tissue injury. On day 6, inflammation was induced by injection of 1 mL of carrageenan 1% (w/v) into the pouches [27, 28]. The carrageenan solution was previously sterilized by autoclaving at 121°C for 15 min. The solution was also supplemented with antibiotics (0.1 mg of penicillin G potassium (Jaber Ebne Hayyan, Iran) and 0.1 mg streptomycin sulfate (Jaber Ebne Hayyan, Iran) per milliliter of the solution) after cooling to 40°C [29]. The inflammatory parameters were determined 72 h after carrageenan injection.

### 2.6. Quantification of Cell Migration and Exudation

Seventy-two hours after injection of the carrageenan solution, the animals were exterminated with an overdose of isoflurane. The pouches were flushed with 3 mL of phosphate buffered saline (PBS) before vigorous massaging for 30 seconds to facilitate further collection of inflammatory cells and exudates. Then, they were opened with a small incision and samples from the air pouch exudate were collected. The amounts of the exudate were quantified and inflammatory cells were counted for determination of total leukocytes. Total leukocyte counts were performed in a Neubauer chamber by diluting the exudate in PBS solution [28].

### 2.7. Determination of TNF*α*, PGE_2_, and VEGF Concentrations in the Pouch Fluid

Injection of carrageenan induces inflammation. The pouch serves as a reservoir of mediators, the levels of which can be easily measured in the fluid that accumulates in the region. Seventy-two hours after the injection of carrageenan solution, the rats were sacrificed by administering an overdose of isoflurane. The pouch was opened with a small incision and the exudate was collected. The fluid contained in the pouch was centrifuged at 10,000 g for 10 min to remove any residual infiltrating leukocytes. The levels of TNF*α*, PGE_2_, and VEGF in the supernatants yielded were measured using commercial ELISA kits (Invitrogen Company, USA, Cusabio Biotech Company, China, and Biospes Company, China, resp.) according to the manufacturer's instructions.

### 2.8. Determination of Granulation Tissue Weight

The procedure of air pouch model of inflammation stimulates the proliferation of cells that cover the surface of the cavity to form a structure similar to that of the synovial fluid [30]. Injection of air over a period of 6 days produced a lining of granulation tissue. Three days after injection of the carrageenan solution, granulation tissue was formed. The tissue was dissected and weighed.

### 2.9. Determination of Angiogenesis in Granulation Tissue

Measurement of angiogenesis in granulation tissue was conducted according to the methods described by Ghosh et al. [31] with some slight modifications. Three days after injection of carrageenan solution, the dissected granulation tissue was washed in PBS (pH = 7.4) and was cut into small pieces using scissors before being homogenized in 3 mL of Drabkin reagent (ZiestChem Diagnostics, Iran) using a homogenizer (HO4 AP-Edmund Bühler, B. Braun, Germany) for 4 min at scale 40 on an ice bed. The tissue homogenate was centrifuged at 10,000 g at 4°C for 30 min. The supernatants were filtered through a 0.22 *μ*m filter (Millipore, Germany). Hemoglobin concentration in the supernatant was then spectrophotometrically determined by measuring absorbance at 540 nm using a hemoglobin assay kit (hemoglobin colorimetric-method, ZiestChem Diagnostics). The amount of hemoglobin in the granulation tissue was expressed as mg hemoglobin/100 g wet tissue.

### 2.10. Statistical Analysis

All results were expressed as mean ± SEM. Statistical significance differences between groups were investigated by one way analysis of variance (ANOVA) followed by least significance difference (LSD) posttest for multiple comparisons. Differences between groups were considered to be statistically significant at a level of *P* < 0.05.

## 3. Results

### 3.1. Free Radical Scavenging Activity of* Ficus carica *Leaves Methanolic Extract

To date, there is no published data on the antioxidant properties of* Ficus carica* harvested from East Azerbaijan; the first aim of this study was to determine the antioxidant activity of* Ficus carica* leaves methanolic extract. The DPPH method adopted for determining free radical scavenging activity of* Ficus carica* is a widely used method for determining the scavenging activity of plant extracts. Antioxidants are substances which reduce the radical form of DPPH through donation of electron or hydrogen; by this reaction, DPPH changes the color of the solution from purple to yellow. The scavenging activity of* Ficus carica* leaves extract is indicated as RC_50_ with lower RC_50_ indicating stronger antioxidant capacity. When compared to a standard antioxidant quercetin (RC_50_: 0.0039 mg/mL), the RC_50_ of methanolic extract of* Ficus carica* leaves was 0.0903 mg/mL.

### 3.2. Total Phenolic Content of* Ficus carica *Leaves Methanolic Extract

The total phenolic content was determined by comparing with standard gallic acid. The total phenolic content of* Ficus carica* methanolic extract was 11.696 mg gallic acid equivalent (GAE)/100 g dry methanolic extract.

### 3.3. The Effects of* Ficus carica* Leaves Methanolic Extract, Diclofenac, and Dexamethasone on Cellular Infiltration, Exudation, and Granulomatous Tissue Weight

Nine days following administration of different doses of methanolic extract of* Ficus carica* leaves, inflammatory parameters such as volume of exudate and leukocytes number were investigated. Analysis of the inhibitory profiles using the investigated doses (5, 25, and 50 mg/pouch) of methanolic extract of* Ficus carica* leaves indicated that significant inhibition of leukocyte migration occurred (by 49.5% (*P* < 0.05), 74.5% (*P* < 0.01), and 76.5% (*P* < 0.01) for each dose, resp.) (Figure 1(a)). The volumes of exudate were also significantly decreased with increasing doses of the extract (Figure 1(b)). As expected, intrapouch administration of diclofenac (1.0 mg/kg) and dexamethasone (0.4 mg/kg) as positive controls caused significant inhibition of leukocytes and exudation.

The anti-inflammatory effects of* Ficus carica* extract in three different doses (5, 25, and 50 mg/pouch) on the granulomatous tissue weight were 28.8%, 25.0%, and 46.2%, respectively. The extract (50 mg/pouch) produced higher reduction in granulomatous tissue weight when compared to the other doses (*P* < 0.05). In addition, the inhibitory effects of 50 mg/pouch exerted on tissue weight seemed to be similar as that reported for diclofenac (36.2%, *P* > 0.05) but were worse than that reported for dexamethasone (81.7%, *P* < 0.001) (Figure 1(c)).

**Figure 2:**
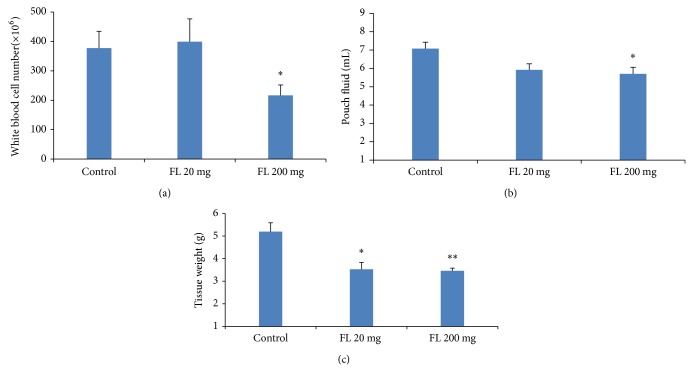
Investigation on the effects of* Ficus carica* extract (a) on the total number of leukocytes in the pouch fluid and (b) pouch fluid volume and (c) granulation tissue weight, 72 h after carrageenan injection for the different groups. Values are the mean with SEM shown by vertical bars. Asterisks indicate significant difference when compared to the control; ^∗^ *P* < 0.050, ^∗∗^ *P* < 0.010 based on ANOVA; FL:* Ficus carica.*

For the investigation on intraperitoneal administration of the extract, only 200 mg of the extract significantly decreased leukocytes and exudation (*P* < 0.05, Figures 2(a) and 2(b)) even though both doses significantly decreased tissue weights (*P* < 0.05 and *P* < 0.01, resp.) (Figure 2(c)). Based on these results, the inhibitory effects of the dose 200 mg of extract on white blood cell count (with no exudation or increase in tissue weight seen) were significantly higher than that for the 20 mg of extract (*P* < 0.01).

### 3.4. The Effects of Methanolic Extract of* Ficus carica* Leaves and Diclofenac on the Angiogenesis and VEGF Levels

**Figure 3:**
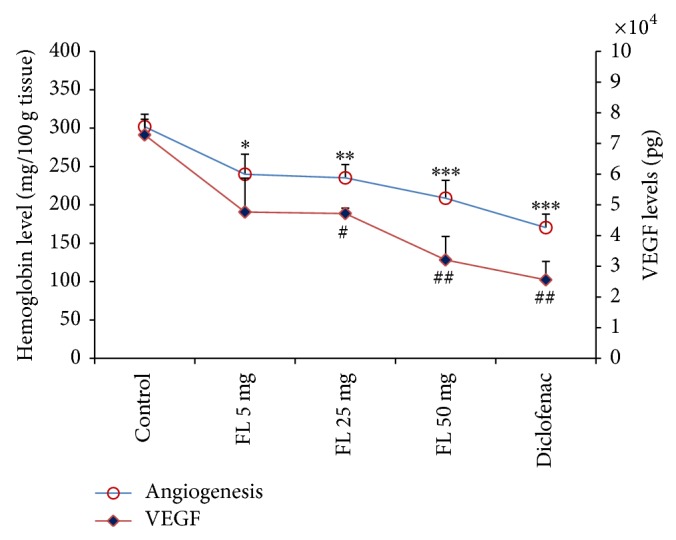
Investigation on the effects of methanolic extract of* Ficus carica* leaves on hemoglobin level as angiogenesis marker and VEGF level 72 h following carrageenan injection. Values are the mean ± SEM shown by vertical bars. Asterisks indicate significant change from control; ^∗^ *P* < 0.050, ^∗∗^ *P* < 0.010, and ^∗∗∗^ *P* < 0.001; ^#^ *P* < 0.050 and ^##^ *P* < 0.010; FL:* Ficus carica*.

There was a significant decrease in hemoglobin content as an angiogenesis marker since* Ficus carica* methanolic extract significantly decreased hemoglobin levels when compared to control rats for all three doses (Figure 3). Similar to diclofenac which decreased VEGF levels, the level of inflammatory exudates was also decreased by the extracts with the reduction seen in a dose-dependent manner (by 34.5%, 35.2%, and 56.0% for 5, 25, and 50 mg/pouch, resp.) (Figure 3). Interestingly, the reduction of angiogenesis and VEGF by methanolic extract of* Ficus carica* leaves (50 mg/pouch) was almost similar to that for diclofenac sodium.

Similarly, for intraperitoneal administration, the extract also significantly (*P* < 0.01) decreased hemoglobin levels to 226.8 ± 14.8 mg/100 g tissue. However, this is only true for the 200 mg doses (figure not shown). For VEGF levels, there were no significant changes among the* Ficus carica*-treated group.

### 3.5. The Effects of Methanolic Extract of* Ficus carica* Leaves, Diclofenac, and Dexamethasone on Cytokine Levels

To investigate the modulatory effect of* Ficus carica* extract on the overall effects of inflammatory cytokines, the production of PGE_2_ and TNF*α* levels was determined. The animals were treated with extract or vehicle just before carrageenan injection and then once a day on two consecutive days. Then, PGE_2_ and TNF*α* levels were measured in the inflammatory exudates extracted from the air pouches 72 h after induction of inflammation.

**Figure 4:**
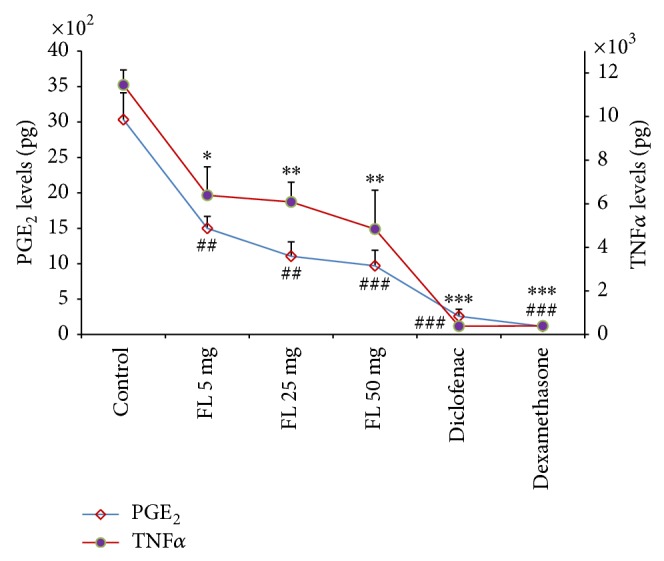
The effects of methanolic extract of* Ficus carica *leaves on PGE_2_ and TNF*α* levels. Values are mean ± SEM shown by vertical bars. Asterisks indicate significant change from control; ^∗^ *P* < 0.050, ^∗∗^ *P* < 0.010, and ^∗∗∗^ *P* < 0.001; ^#^ *P* < 0.050 and ^##^ *P* < 0.010; FL:* Ficus carica*.

Figure 4 depicts the dose-dependent variations in PGE_2_ and TNF*α* concentrations in response to increasing concentrations of the extract. Intrapouch injection of the extracts (5, 25, and 50 mg/pouch) again decreased PGE_2_ levels by 50.6%, 63.6%, and 68%, respectively. Similarly, significant reduction in TNF*α* concentrations was also achieved following administration of the extracts (5, 25, and 50 mg/pouch) by 44.2%, 46.9%, and 57.8%, respectively. However, the percentage of PGE_2_ and TNF*α* reductions achieved by diclofenac (1 mg/pouch) and dexamethasone (0.4 mg/pouch) is higher (91.5%, 96.2% and 96.7%, 96.6%, resp.) (Figure 4).

## 4. Discussion

Increasing awareness of the effectiveness of herbal medicines, besides the apparent side effects of synthetic drugs, leads to an increased interest in herbal medicines [32]. The present study established the anti-inflammatory and antiangiogenesis activities of methanolic extract of* Ficus carica* leaves in the models used. These effects are very important, because angiogenesis is a leading cause of cancer and chronic inflammatory conditions such as RA besides being central to maintaining and promoting RA. Several recent studies in animal models of arthritis have suggested that blocking angiogenesis during the course of RA is therapeutically beneficial [33]. Our findings strongly indicated that treatment with the extract reduced angiogenesis in the rats' granulomatous tissue.

Consistent with our findings, Mostafaie et al. also found that* Ficus carica* latex extract had antiangiogenic and antiproliferative effects in the human umbilical vein endothelial cells model in a dose-dependent manner [34]. However, unlike our study which was conducted* in vivo*, their investigation was conducted* in vitro* and they have focused on the extract of the latex. Nevertheless, further investigation on the mechanism of action of* Ficus carica* has to be conducted to confirm both findings.

Having important role in tissue injury and inflammation, the free radical scavenging capacity of* Ficus carica* leaves extract was measured by DPPH assay. We measured the concentration of stable free radical DPPH at 517 nm which indicated the strength of the antioxidant substance. Low DPPH concentrations indicate a stronger antioxidant activity [35] as seen in the methanolic extract of* Ficus carica* leaves.

Recent studies have shown that free radical scavenging activity of medicinal plants is responsible for their anti-inflammatory effect [36, 37]. In our study, we found that the* Ficus carica* methanolic extract also contained high total phenolic content indicating its high antioxidant properties. Overall, taking together the findings from the DPPH inhibition and total phenolic content, our study indicated that* Ficus carica* extract has significant antioxidant activity. Therefore, it is plausible that the anti-inflammatory and antiangiogenesis properties of the extract are contributed by the high antioxidant capacity shown.

Numerous researchers found that ethanolic extract of* Ficus carica* leaves possesses a significant antipyretic effect with comparable effects to that of paracetamol. Fever may be a result of infection or a result of one of the sequels of tissue damage, inflammation, graft rejection, or other disease states [1]. In addition, another group of researchers [38] has shown that the presence of flavonoids and polyphenols is the basis for the analgesic and anti-inflammatory activities of various parts of* Ficus carica* including the fruit, latex, bark, roots, and leaves. In addition, the total phenolic content is significantly different among the different vegetal parts with the leaves reported to contain the highest levels. The methanolic extract of the leaves has also been shown to exhibit the highest antioxidant potential [39] and therefore the leaves were utilized in this study.

In this study, the anti-inflammatory effects of the extract were investigated including the total number of leukocytes, exudate volume, tissue weight, inflammatory mediators, and angiogenesis. Pharmacological and chemical studies have also demonstrated antineoplastic or anti-inflammatory activity of both the crude extract and pure compounds [40]. The observed strong antioxidant activity of* Ficus carica* may be due to the presence of several phenolic compounds such as flavonoids [41], the presence of which was confirmed in our study.

Inflammation is increasingly recognized as a critical mediator of angiogenesis and unregulated angiogenic response has been reported to be involved in chronic inflammation [19]. Angiogenesis is involved in RA leading to leukocyte recruitment and inflammation in the synovium. Furthermore, synovial inflammation itself further potentiates endothelial proliferation and angiogenesis [15].

To elucidate the mechanism of anti-inflammatory and antiangiogenesis effects of* Ficus carica* extract, the concentrations of a number of candidate proinflammatory mediators were measured. Our results clearly indicated that the extract reduced PGE_2_, TNF*α*, and VEGF levels in the inflammatory exudates. Therefore, it may have the potential to exert antirheumatic effects due to its ability to downregulate proinflammatory cytokines. In another study, Patil et al. (2011) [42, 43] also found that* Ficus carica* extract possesses anti-inflammatory effects. However, as opposed to the ethanolic extract, they have found that the petroleum ether and chloroform extracts failed to show any anti-inflammatory effects perhaps due to the absence of antioxidant contents in these extracts indicating that the type of solvent used is crucial. In another study,* Ficus carica* fruit ethanolic extract was also shown to have anti-inflammatory effects [42, 43].

Another point to consider is the fact that the air pouch model has been reported to have histological similarity to synovial membranes and when challenged via an injection of carrageenan the inflammatory reaction is histologically similar to that observed in the chronic synovial inflammation [27]. Thus, in the present study, the reduction in inflammatory parameters seen in the air pouch model induced by* Ficus carica* extract is in agreement with the studies of Park et al. [44] in osteoclastogenesis in cell culture and in bone marrow-derived macrophages which further supports the role of* Ficus carica* extract in RA.

Patients' joint lesions with RA are characterized by synovial hyperplasia with an increased number of fibroblasts and infiltrating immune cells. Synovial exudates in RA are abundant in neutrophils, monocytes, macrophages, and lymphocytes which release degradative enzymes, PGs, reactive oxygen species, and proinflammatory cytokines which contribute to the pathogenesis of the disease. The migration of cells into the inflamed joint is mediated by chemokines released by the activated cells in the joint. Evidence indicates that accumulation of chemokines in the synovial tissue contributes to the pathogenesis of synovitis [45]. Our findings indicated that pretreatment of the animals with* Ficus carica* extract inhibits the production of proinflammatory cytokines including TNF*α* and PGE_2_ that cause joint injury by inducing activation and recruitment of leukocytes and exudation at the site of inflammation in experimental model of arthritis. Therefore,* Ficus carica* has the potential to be used in the treatment of RA.

It is now accepted that TNF*α* is an angiogenic factor [46]. In addition to the TNF*α* pathway,* Ficus carica* extract exerted inhibitory activity on the* in vivo* release of PGE_2_, which is produced from arachidonic acid via cyclooxygenase-2 (COX-2) [19]. The data reported by Ghosh et al. study [47] indicates that COX-2-derived PGE_2_ plays a significant role in angiogenesis in the carrageenan-induced granulation tissue through VEGF formation. Therefore, it is plausible that* Ficus carica* extract directly inhibits COX-2 or deactivates inflammatory cells expressing COX-2.

Another question addressed in this study is whether there is a differential effect on inflammation by administering the extract locally versus systemically. In this study, we demonstrated that, in chronic inflammation, the administration of* Ficus carica* extract directly into the inflamed site decreased inflammatory response. Therefore, it may be concluded that inhibiting PGE_2_, TNF*α*, and VEGF generations which tend to occur locally may account for the anti-inflammatory response.

It is interesting to note that the inhibitory properties presented by the extract in this work have some similarities with the anti-inflammatory effects produced by diclofenac sodium suggesting that they share similar anti-inflammatory pathways. Further studies to elucidate the exact mechanism of action of* Ficus carica* in the inflammatory conditions are recommended. In addition, our findings should be further confirmed pathologically using histopathological examination.

## 5. Conclusion

*Ficus carica* methanolic extract has potent anti-inflammatory activities at the level of cell migration, exudate volume, tissue weight, and angiogenesis as well as in the content of proinflammatory mediators such as PGE_2_, TNF*α*, and VEGF levels which were more pronounced when it was injected locally as opposed to systemically.

## References

[1] Vikas V. P., Bhangale S. C., Patil V. R. (2010). Evaluation of anti-pyretic potential of *Ficus carica* leaves. *International Journal of Pharmaceutical Sciences Review and Research*.

[2] Trad M., Ginies C., Gaaliche B., Renard C. M. G. C., Mars M. (2012). Does pollination affect aroma development in ripened fig [*Ficus carica* L.] fruit?. *Scientia Horticulturae*.

[3] Veberic R., Colaric M., Stampar F. (2008). Phenolic acids and flavonoids of fig fruit (*Ficus carica* L.) in the northern Mediterranean region. *Food Chemistry*.

[4] Çalişkan O., Aytekin Polat A. (2011). Phytochemical and antioxidant properties of selected fig (*Ficus carica* L.) accessions from the eastern Mediterranean region of Turkey. *Scientia Horticulturae*.

[5] Kvien T. K. (2004). Epidemiology and burden of illness of rheumatoid arthritis. *PharmacoEconomics*.

[6] Dalmarco E. M., Astolfi G., de Liz R., de Córdova C. M. M., Fröde T. S. (2012). Modulatory effect of mycophenolate mofetil on carrageenan-induced inflammation in the mouse air pouch model. *International Immunopharmacology*.

[7] Merlino L. A., Curtis J., Mikuls T. R., Cerhan J. R., Criswell L. A., Saag K. G. (2004). Vitamin D intake is inversely associated with rheumatoid arthritis: results from the Iowa Women's Health Study. *Arthritis and Rheumatism*.

[8] Garella S., Matarese R. A. (1984). Renal effects of prostaglandins and clinical adverse effects of nonsteroidal anti-inflammatory agents. *Medicine*.

[9] Colville-Nash P. R., Scott D. L. (1992). Angiogenesis and rheumatoid arthritis: pathogenic and therapeutic implications. *Annals of the Rheumatic Diseases*.

[10] Martin S. W., Stevens A. J., Brennan B. S., Davies D., Rowland M., Houston J. B. (1994). The six-day-old rat air pouch model of inflammation: characterization of the inflammatory response to carrageenan. *Journal of Pharmacological and Toxicological Methods*.

[11] Esser R. E., Miserendino-Molteni R., Sharr M. (2005). Pharmacodynamic behaviour of the selective cyclooxygenase-2 inhibitor lumiracoxib in the lipopolysaccharide-stimulated rat air pouch model. *European Journal of Pharmaceutical Sciences*.

[12] Carmeliet P. (2003). Angiogenesis in health and disease. *Nature Medicine*.

[13] Paleolog E. M. (2002). Angiogenesis in rheumatoid arthritis. *Arthritis Research*.

[14] Clavel G., Bessis N., Boissier M.-C. (2003). Recent data on the role for angiogenesis in rheumatoid arthritis. *Joint Bone Spine*.

[15] Clavel G., Valvason C., Yamaoka K. (2006). Relationship between angiogenesis and inflammation in experimental arthritis. *European Cytokine Network*.

[16] Makhni E. (2003). Angiogenesis: an examination of both tumorigenic and rehabilitative properties. *MIT Undergraduate Research Journal*.

[17] Kubota Y. (2012). Tumor angiogenesis and anti-angiogenic therapy. *Keio Journal of Medicine*.

[18] Clark I. A. (2007). How TNF was recognized as a key mechanism of disease. *Cytokine & Growth Factor Reviews*.

[19] Zhang Y., Daaka Y. (2011). PGE2 promotes angiogenesis through EP4 and PKA C*γ* pathway. *Blood*.

[20] Namkoong S., Lee S.-J., Kim C.-K. (2005). Prostaglandin E2 stimulates angiogenesis by activating the nitric oxide/cGMP pathway in human umbilical vein endothelial cells. *Experimental and Molecular Medicine*.

[21] Form D. M., Auerbach R. (1983). PGE2 and angiogenesis. *Proceedings of the Society for Experimental Biology and Medicine*.

[22] Leahy K. M., Koki A. T., Masferrer J. L. (2000). Role of cyclooxygenases in angiogenesis. *Current Medicinal Chemistry*.

[23] Renz H., Gong J.-H., Schmidt A., Nain M., Gemsa D. (1988). Release of tumor necrosis factor-*α* from macrophages: Enhancement and suppression are dose-dependently regulated by prostaglandin E_2_ and cyclic nucleotides. *Journal of Immunology*.

[24] Appleton I., Brown N. J., Willis D. (1996). The role of vascular endothelial growth factor in a murine chronic granulomatous tissue air pouch model of angiogenesis. *The Journal of Pathology*.

[25] Ferrara N., Gerber H.-P. (2001). The role of vascular endothelial growth factor in angiogenesis. *Acta Haematologica*.

[26] Heshmati-Afshar F., Delazar A., Nazemiyeh H., Esnaashari S., Moghadam S. B. (2012). Comparison of the total phenol, flavonoid contents and antioxidant activity of methanolic extracts of *Artemisia spicigera* and *A. splendens* growing in Iran. *Pharmaceutical Sciences*.

[27] Sedgwick A. D., Sin Y. M., Edwards J. C. W., Willoughby D. A. (1983). Increased inflammatory reactivity in newly formed lining tissue. *The Journal of Pathology*.

[28] Duarte D. B., Vasko M. R., Fehrenbacher J. C. (2012). Models of inflammation: carrageenan air pouch. *Current Protocols in Pharmacology*.

[29] Ghosh A. K. (2003). Regulation by prostaglandin E2 and histamine of angiogenesis in inflammatory granulation tissue. *Yakugaku Zasshi*.

[30] Edwards J. C. W., Sedgwick A. D., Willoughby D. A. (1981). The formation of a structure with the features of synovial lining by subcutaneous injection of air: an in vivo tissue culture system. *Journal of Pathology*.

[31] Ghosh A. K., Hirasawa N., Ohtsu H., Watanabe T., Ohuchi K. (2002). Defective angiogenesis in the inflammatory granulation tissue in histidine decarboxylase-deficient mice but not in mast cell-deficient mice. *The Journal of Experimental Medicine*.

[32] Ghorbani A. (2014). Clinical and experimental studies on polyherbal formulations for diabetes: current status and future prospective. *Journal of Integrative Medicine*.

[33] Paleolog E. M. (2002). Angiogenesis in rheumatoid arthritis. *Arthritis Research*.

[34] Mostafaie A., Mansouri K., Norooznezhad A.-H., Mohammadi-Motlagh H.-R. (2011). Anti-angiogenic activity of *Ficus carica* latex extract on human umbilical vein endothelial cells. *Cell Journal*.

[35] Delazar A., Shoeb M., Kumarasamy Y. (2004). Two bioactive ferulic acid derivatives from Eremostachys glabra. *DARU Journal of Pharmaceutical Sciences*.

[36] Konyalioglu S., Saglam H., Kivçak B. (2005). *α*-tocopherol, flavonoid, and phenol contents and antioxidant activity of *Ficus carica*. leaves. *Pharmaceutical Biology*.

[37] Cuzzocrea S., Riley D. P., Caputi A. P., Salvemini D. (2001). Antioxidant therapy: a new pharmacological approach in shock, inflammation, and ischemia/reperfusion injury. *Pharmacological Reviews*.

[38] Modi R. K., Kawadkar M., Sheikh S., Kastwar R., Tiwari G. (2012). A review on: comparative studies on ethanolic extract of root and stem bark of *Ficus carica* for analgesic and antiinflammatory activities. *International Journal of Pharmacy & Life Sciences*.

[39] Sirisha N., Sreenivasulu M., Sangeeta K., Madhusudhana Chetty C. (2010). Antioxidant properties of Ficus Specie—a review. *International Journal of PharmTech Research*.

[40] Lansky E. P., Paavilainen H. M., Pawlus A. D., Newman R. A. (2008). *Ficus* spp. (fig): ethnobotany and potential as anticancer and anti-inflammatory agents. *Journal of Ethnopharmacology*.

[41] Saoudi M., El Feki A. (2012). Protective role of Ficus carica stem extract against hepatic oxidative damage induced by methanol in male Wistar rats. *Evidence-Based Complementary and Alternative Medicine*.

[42] Patil V. V., Patil V. R. (2011). Evaluation of anti-inflammatory activity of *Ficus carica* Linn. leaves. *Indian Journal of Natural Products and Resources*.

[43] Koka S., Barik R., Joshi J., Jain S. (2013). Effect of *Ficus carica* fruit extract on experimentally induced inflammation and nociception. *Journal of Pharmacy and Phytotherapeutics*.

[44] Park Y. R., Eun J. S., Choi H. J. (2009). Hexane-soluble fraction of the common fig, Ficus carica, inhibits osteoclast differentiation in murine bone marrow-derived macrophages and RAW 264.7 cells. *Korean Journal of Physiology and Pharmacology*.

[45] Tani K., Shimizu T., Motoki Y., Sone S. (2002). Chemokines in synovial inflammation in rheumatoid arthritis: basic and clinical aspects. *Modern Rheumatology*.

[46] Naldini A., Carraro F. (2005). Role of inflammatory mediators in angiogenesis. *Current Drug Targets: inflammation and Allergy*.

[47] Ghosh A. K., Hirasawa N., Niki H., Ohuchi K. (2000). Cyclooxygenase-2-mediated angiogenesis in carrageenin-induced granulation tissue in rats. *Journal of Pharmacology and Experimental Therapeutics*.

